# Diagnostic Accuracy of the Truenat MTB Plus Assay and Comparison with the Xpert MTB/RIF Assay to Detect Tuberculosis among Hospital Outpatients in Cameroon

**DOI:** 10.1128/jcm.00155-22

**Published:** 2022-07-21

**Authors:** Yannick Russel Ngangue, Cyrille Mbuli, Angela Neh, Emmanuel Nshom, Armand Koudjou, Dennis Palmer, Norah Nyah Ndi, Zhi Zhen Qin, Jacob Creswell, Vincent Mbassa, Comfort Vuchas, Melissa Sander

**Affiliations:** a Center for Health Promotion and Research, Bamenda, Cameroon; b Cameroon Baptist Convention Health Services, Bamenda, Cameroon; c Baptist Institute of Health Sciences, Bamenda, Cameroon; d Stop TB Partnership, Geneva, Switzerland; e National TB Program, Yaounde, Cameroon; University of Manitoba

**Keywords:** *Mycobacterium tuberculosis*, diagnostics, molecular methods

## Abstract

The Truenat MTB Plus assay is a rapid molecular test that has been recommended by the World Health Organization since 2020 as an initial test to detect tuberculosis (TB). The WHO highlighted the need to further evaluate assay performance to inform future recommendations, including in people living with HIV and compared to the Xpert MTB/RIF assay. We conducted a prospective evaluation of the diagnostic accuracy of the Truenat assay in Cameroon, a country with a high burden of HIV/TB. Adult outpatients were recruited at four hospitals; demographic information and medical history were collected, and participants produced two sputum specimens. Truenat and Xpert testing was performed on the same specimen, and performance was compared to TB culture as the reference standard. From November 2019 to December 2020, 945 participants were enrolled and included in the analysis. Among 251 participants with culture-positive TB, the sensitivity of Truenat MTB Plus was 91% (95% confidence interval [CI], 86 to 94%), similar to Xpert (90%; 95% CI, 86 to 93%). Among 74 HIV-positive participants with culture-positive TB, the sensitivity of Truenat MTB Plus was 85% (95% CI, 75 to 92%) compared to 81% for Xpert (95% CI, 70 to 89%). Among 47 participants with smear-negative TB, the sensitivity of Truenat MTB Plus was 55% (95% CI, 40 to 70%), similar to Xpert (53%; 95% CI, 38 to 68%). The specificity of Truenat MTB Plus was 96% (95% CI, 94 to 97%) compared to 99% (95% CI, 97 to 99%) for Xpert. For TB detection compared to the reference standard of TB culture, the performance of the Truenat MTB Plus assay was similar to that of Xpert in this population, including among people living with HIV.

## INTRODUCTION

Tuberculosis (TB) is a significant global health challenge, with an estimated 10 million people becoming sick with the disease and 1.5 million deaths due to TB in 2020 (1). The global public health response for TB has been complicated by challenges to diagnose and link people to care. In 2019, an estimated 2.9 million people with TB were undiagnosed and unreported (2), and in 2020 this estimate increased to more than 4 million people undiagnosed and unreported for TB during the COVID-19 pandemic (1). Ensuring that better diagnostic tools are developed and made accessible to people to be evaluated for the disease are key priorities for ending TB (3). Rapid, inexpensive, and sensitive point-of-care tests that can be used to diagnose active TB disease from more readily obtainable, nonsputum specimens are in development and may greatly improve TB elimination efforts (4, 5). However, such tests are not yet widely available, and at present, diagnostic testing for TB is still typically sputum and hospital based.

The currently recommended algorithms to diagnose TB include the use of molecular-based tests to detect TB and drug-resistant TB (6, 7). The Xpert MTB/RIF assay (Cepheid, USA), a rapid molecular diagnostic tool for TB, was recommended by the World Health Organization (WHO) in 2010, and the Xpert MTB/RIF Ultra assay, a more sensitive test, was recommended in 2017. Use of the GeneXpert system with both the Xpert and Ultra assays has been widely scaled up; however, uptake in some settings has been hindered by issues such as the relatively high cost of the tests, lack of availability of the required infrastructure needs for the equipment, and technical challenges to keep the instruments running (8, 9). Until 2020, the Xpert system was the only WHO-recommended option for rapid molecular detection of TB and rifampin-resistant TB; however, it is beneficial for end-users to have access to multiple options for diagnostic testing (10).

The TrueNat MTB Plus assay (Molbio Diagnostics, India) is a more recently available molecular test that runs on the portable, battery-operated Truenat platform and, to date, has been used primarily in India (11–16). In 2020, the WHO recommended the TrueNat MTB or MTB Plus assay as the initial diagnostic test for TB rather than smear microscopy/culture, along with the Truenat MTB-RIF Dx for detection of rifampin resistance in people with a positive Truenat MTB or MTB Plus result (17). This recommendation followed a multicenter evaluation of the Truenat assays conducted by the Foundation for Innovative New Diagnostics (FIND) at seven sites in four countries (18). The WHO has highlighted the need for additional evaluations of the diagnostic accuracy of these assays in a variety of settings and populations, including for people living with HIV, to inform future recommendations (6).

Cameroon has a high burden of TB/HIV, and in 2020, only an estimated 48% of people with TB were diagnosed and treated for the disease (1). The country has an estimated TB incidence of 174 per 100,000 people, an HIV prevalence among adults of 2.7% (19), and an HIV/TB incidence of 42/100,000 (1). Rapid, sensitive tests for TB detection have the potential to greatly improve TB diagnosis and care in this setting.

In this study, we assessed the sensitivity and specificity of the Truenat MTB Plus assay compared with the Xpert MTB/RIF assay and against TB culture as a reference standard. This evaluation was performed on sputum samples collected from ambulatory adults presenting with TB symptoms at four hospitals in Cameroon.

## MATERIALS AND METHODS

### Setting and study design.

This was a prospective, multisite, diagnostic accuracy study. The primary objective was to evaluate the sensitivity and specificity of the Truenat MTB Plus to detect Mycobacterium tuberculosis complex compared to the reference standard of TB culture. The secondary objective was to compare the sensitivity and specificity of the Truenat MTB Plus assay with those of the Xpert MTB/RIF assay, which was the assay most widely used in Cameroon at the time of the study. While the Truenat MTB Plus is designed to be used with the Truenat MTB RIF Dx assay to detect rifampin resistance in people with TB, we did not include an evaluation of the performance of the MTB RIF Dx assay to detect rifampin-resistant TB as part of this work. The study was conducted in four hospitals in three regions of the country: the Mbingo Baptist Hospital and the Nkwen Baptist Health Center (Northwest region), the Mutengene Baptist Hospital (Southwest region), and the Mboppi Baptist Hospital (Littoral region). Laboratory testing was performed at the Tuberculosis Reference Laboratory Bamenda, which is accredited in accordance with the recognized International Standard ISO 15189:2012 (SANAS Accredited Medical Laboratory, no. M0593). This study was approved by the Institutional Review Board of the Cameroon Baptist Convention Health Board (IRB2019-26). All participants provided written informed consent.

### Participants.

Consecutive people aged 15 years of age and older who were referred for TB testing to the laboratories of any of the four study sites were screened for eligibility. People with a prolonged cough of at least 2 weeks and any one or more of fever, night sweats, and/or weight loss were invited to participate. Individuals who were currently on TB treatment or who reported having taken any TB treatment within the previous 6 months were excluded. After providing written informed consent, a questionnaire was administered to the participants, including details of any previous TB treatment history. Anyone not known to be HIV positive and without a negative HIV result within the previous 1 month was counseled and offered HIV testing, following routine practice at the study hospitals. Participants were contacted at 2 months postenrollment to document their clinical and TB treatment status. Individual participant data are available as supplemental material (see Data Set S1 in the supplemental material).

### Specimen collection.

All participants were instructed on how to produce two sputum specimens with a volume of at least 4 mL each. Participants were asked to collect sputum into 50-mL tubes until this volume was reached; in cases where the volume of sputum was not sufficient, the specimens were processed and testing results provided to the clinicians as usual, but the participant was considered as an early exclusion and the data were not included in the analysis. In some cases, the first specimen was produced on the spot and the second specimen was produced in the early morning on a subsequent day. If the participant could not expectorate a spot specimen, then a first morning specimen and a subsequent second specimen was collected on the spot or as a second morning specimen as possible. Specimens were stored at 2 to 8°C and transported to the reference laboratory, where all laboratory testing was performed.

### Laboratory methods.

Sputum specimens of >4 mL were initially homogenized by vortexing the specimens with 3-mm sterile glass beads for 20 min. Then, the homogenized sputum was aliquoted for direct microscopy (50 μL), culture processing (1.25 mL), Truenat testing (0.5 mL), and Xpert testing (0.5 mL), and the remainder was stored for additional testing as needed.

### (i) Reference standard.

For each homogenized sputum specimen, a direct microscopy smear with approximately 50 μL sputum was prepared and examined for acid-fast bacilli (AFB) by fluorescence microscopy. Then, 1.25 mL of sputum was processed using the *N*-acetyl-l-cysteine–NaOH (NALC-NaOH) method for TB culture (20). After centrifugation, the pellet was resuspended in the same volume as the starting sputum (1.25 mL) of phosphate-buffered saline (PBS) buffer; then, 0.5 mL was inoculated on mycobacterial growth indicator tubes (MGIT; BD Diagnostic Systems, Sparks, MD, USA) using the Bactec MGIT 960 system, and ~0.2 mL was inoculated on Lowenstein-Jensen media. Cultures positive for acid-fast bacilli were tested for M. tuberculosis complex by MPT64 antigen detection (Standard Diagnostics, Republic of Korea). Isolates that were AFB positive and negative by MPT64 antigen testing were tested for mycobacterial identification using the GenoType Mycobacterium CM and AS line probe assays (Hain Lifescience, Germany) according to the manufacturer’s instructions. Cultures were read without reference to other diagnostic test results.

### (ii) Index test.

A total of 0.5 mL of homogenized sputum was added to 3 mL specimen reagent (lysis buffer) for nucleic acid extraction on the Trueprep Auto Universal cartridge (Molbio, India). The elute obtained was tested using the Truenat MTB Plus assay (Molbio, India) following the manufacturer’s instructions (21). The Truenat MTB Plus assay produces results of invalid, error, MTB (M. tuberculosis complex) not detected, or MTB detected with a grade of high, medium, low, or very low.

### (iii) Comparator test.

A total of 0.5 mL of homogenized sputum was added to 1.5 mL of the Xpert specimen reagent and tested using the Xpert MTB/RIF assay (Cepheid, Sunnyvale, CA, USA) following the manufacturer’s instructions. The Xpert MTB/RIF assay produces results of invalid, error, MTB not detected, or MTB detected with a grade of high, medium, low, or very low.

### (iv) Review of discordant results.

For specimens that tested positive on the Truenat MTB Plus and had negative results by the reference standard of culture, sputum was also tested on the Xpert MTB/RIF Ultra assay. This testing was performed to evaluate whether TB was also detected using this assay, which has a higher sensitivity than the comparator Xpert MTB/RIF assay.

### Data analysis.

**FIG 1:**
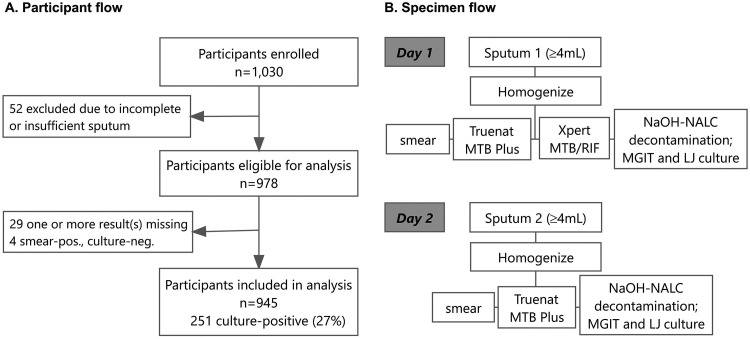
Flow of people in study (A), and flow of specimens in study (B). For specimen testing at the reference laboratory, each sputum specimen of ≥4 mL was homogenized, and then ~50 μL was aliquoted and tested by direct smear microscopy, 0.5 mL was aliquoted and tested for each of the Xpert MTB/RIF and Truenat MTB Plus assays, and 0.5 mL of the resuspended pellet after sputum processing was inoculated into mycobacterial growth indicator tube (MGIT) liquid culture media, with 0.2 mL inoculated onto Lowenstein-Jensen (LJ) solid culture media; the remaining sputum was stored for retesting or additional testing as needed.

Definitions of TB cases were based on four cultures, one automated liquid culture and one solid culture from each of two sputum specimens, as shown in Fig. 1B. Participants for whom at least one culture was positive for TB were defined as having culture-positive TB; among these, anyone with at least one smear positive for TB was defined as smear positive, while individuals with culture-positive TB who had both smears negative for TB were defined as having smear-negative TB. Participants for whom at least one culture was negative for TB and with no cultures positive for TB were defined as culture negative for TB.

Data from the study files and laboratory registers were double entered and validated by comparison. Participants were characterized using simple descriptive statistics. To evaluate the sensitivity and specificity of the Truenat and Xpert assays, we calculated the point estimates with 95% confidence intervals (CIs) using the Wilson score method (22, 23). We referred to the recent guidance for conducting studies to evaluate the accuracy of sputum-based tests to detect TB for study design and reporting of results (24). The Standards for Reporting of Diagnostic Accuracy (STARD) recommendations were followed for reporting these data (25). Analyses were performed in R version 4.1.2 (26).

### Data availability.

All data used for the analysis are presented in the paper and its supplemental material.

## RESULTS

Participants were enrolled from 19 November 2019 to 2 April 2020 and from 7 July 2020 to 20 December 2020; the study was paused from April to July 2020 due to lockdowns during the COVID-19 pandemic. A total of 1,030 adults with cough >2 weeks and one other symptom who were consecutively referred and presented for laboratory testing at the four hospitals were invited to be enrolled in the study and were screened for TB and HIV, as shown in the participant and specimen flow (Fig. 1). There were 52 participants who, after enrollment, did not provide two specimens or did not provide specimens with sufficient volume (early exclusions). In addition, there were 29 participants with one or more results missing and 4 participants with smear-positive, culture-negative results who were excluded. Data from a total of 945 participants were included in the final analysis.

Of the 945 participants included in the analysis, the median age was 42 years (interquartile range [IQR], 32 to 55), 494 (52%) were female, 352 people (37%) were HIV positive, and 135 (14%) had a history of TB treatment (Table 1). A total of 251 participants (27%) had culture-positive TB, and 47 of these (19%) had smear-negative, culture-positive TB. The majority of participants were enrolled from one hospital (550 participants, 58%), and 174 (32%) participants from this hospital had culture-positive TB.

**TABLE 1:** Clinical and demographic characteristics of 945 participants by tuberculosis culture result

Characteristic	All participants (*n* = 945)^*a*^	TB culture result^*a*^	
		Positive (*n* = 251)	Negative (*n* = 694)
Age, yrs	42 (32–55)	37 (28–47)	44 (34–58)
Female sex	494 (52)	99 (39)	395 (57)
History of TB	135 (14)	24 (10)	111 (16)
HIV infection^*b*^	352 (37)	74 (30)	278 (40)
Hospital			
1	98 (10)	15 (6)	83 (12)
2	108 (11)	15 (6)	93 (13)
3	550 (58)	174 (69)	376 (54)
4	189 (20)	47 (19)	142 (21)

a Data are given as median (interquartile range) or number (%).

b Two participants did not have HIV status recorded.

The sensitivity of the Truenat MTB Plus assay compared to culture for the first sputum specimen collected was 91% (95% CI, 86 to 94%), as shown in Table 2, and was similar to the Xpert sensitivity (90%; 95% CI, 86 to 94%) in this population. Among participants with smear-negative, culture-positive TB, the sensitivity of the Truenat MTB Plus was 55% (95% CI, 40 to 70%) compared to 53% (95% CI, 38 to 68%) with Xpert MTB/RIF.

**TABLE 2:** Sensitivity and specificity of the Truenat MTB Plus assay and Xpert MTB/RIF assay compared to the reference standard of TB culture^*a*^

Assay and demographic	Total no.	No. true positive	No. false positive	No. false negative	No. true negative	Sensitivity % (95% CI)	Specificity % (95% CI)
Truenat MTB Plus							
All participants	945	228	31	23	663	91 (86–94)	96 (94–97)
HIV status^*b*^							
HIV positive	352	63	11	11	267	85 (75–92)	96 (93–98)
HIV negative	591	164	20	12	395	93 (88–96)	95 (93–97)
Smear status^*c*^							
Smear negative	47	26		21		55 (40–70)	
HIV positive	26	15		11		57 (37–76)	
Smear positive	204	202		2		99 (96–100)	
History of TB							
History of TB	135	22	8	2	103	92 (72–99)	93 (86–97)
No history of TB	810	206	23	21	560	91 (86–94)	96 (94–97)
Xpert MTB/RIF							
All participants	945	227	10	24	684	90 (86–94)	99 (97–99)
HIV status^*b*^							
HIV positive	352	60	5	14	273	81 (70–89)	98 (96–99)
HIV negative	591	166	5	10	410	94 (90–97)	99 (97–100)
Smear status^*c*^							
Smear negative	47	25		22		53 (38–68)	
HIV positive	26	12		14		46 (27–66)	
Smear positive	204	202		2		99 (96–100)	
History of TB							
History of TB	135	21	4	3	107	88 (67–97)	96 (90–99)
No history of TB	810	206	6	21	577	91 (86–94)	99 (98–100)

a Results for all participants and by HIV status, smear status (sensitivity), and history of TB, for a total of 945 participants included in the analysis (251 culture positive and 694 culture negative).

b Two participants did not have HIV status recorded.

c Among people with culture-positive TB.

Among the 74 participants with HIV infection and culture-positive TB, the sensitivity of the Truenat MTB Plus was 85% (95% CI, 75 to 92%) and that of Xpert was 81% (95% CI, 70 to 89%). The sensitivity of the Truenat MTB Plus to detect TB in 26 people with HIV infection and smear-negative, culture-positive TB was 57% (15/26; 95% CI, 37 to 76%), while the sensitivity of Xpert in this population was similar (46%, 12/26; 95% CI, 27 to 66%).

Among all participants, the specificity of the Truenat MTB Plus was 96% (95% CI, 94 to 97%) compared to 99% (95% CI, 97 to 99%) for the Xpert MTB/RIF assay. Among participants with a history of TB, the specificity for the Truenat MTB Plus was 93% (95% CI, 86 to 97%) and that for the Xpert MTB/RIF was 96% (95% CI, 90 to 99%). Of the 694 participants without cultures positive for TB, 59 (8.5%) had results of one or two cultures positive for AFB and identified as nontuberculous mycobacteria. The distribution of nontuberculous mycobacteria that were detected are shown in Table S2 in the supplemental material. All of the specimens with cultures identified as nontuberculous mycobacteria had results of TB not detected on Truenat MTB Plus, which is in line with manufacturer’s data that the Truenat MTB Plus assay is highly specific for the detection of Mycobacterium tuberculosis complex (21).

Adding a second Truenat test for a second sputum specimen increased the sensitivity to detect TB among participants with culture-positive TB to 92% (95% CI, 88 to 95%) and decreased specificity among those without culture-positive TB to 93% (95% CI, 91 to 95%), as shown in Table S1 in the supplemental material.

The proportion of Truenat MTB Plus assays with indeterminate results is summarized in Table S3 in the supplemental material. For the lot of Truenat MTB Plus tests used for most tests in this study, 90% (1,217/1,343) of the tests produced valid results on the first amplification attempt; the remaining 10% were a combination of errors (4%) and invalid (6%) results. Of specimens with an indeterminate result on the first attempt, 79% (110/140) had a valid result on the second attempt using leftover extracted nucleic acid. After two attempts, 2% (30/1,343) of specimens had results that were uninterpretable.

Of the 31 participants with a first sputum specimen with a Truenat MTB Plus result of TB detected and with culture results negative for TB (false positives), 74% (23/31) had a second molecular test result positive for TB either on the same specimen or a second specimen from the same participant. Details of individual participant characteristics, TB testing results, and outcomes for these 31 participants are shown in Table S4 in the supplemental material.

Among 299 participants with any molecular and/or culture result with TB detected, all were eligible for treatment, and 274 (92%) started anti-TB treatment, including 4 people who started treatment for multidrug-resistant TB based on confirmed resistance to rifampin. Among the 25 who did not initiate anti-TB treatment, 7 participants died and 10 were lost to follow-up prior to starting treatment, while the remaining 8 did not start treatment for various reasons. Of the 274 participants who started TB treatment, 257 (94%) were alive on treatment at 2 months follow-up, while 7 participants had died (including 5 who were HIV positive), and 10 people were lost to follow-up after starting treatment. Detailed results by individual are shown in the full data set (see Data Set S1 in the supplemental material).

## DISCUSSION

In this study of 945 people with symptoms of TB conducted in four hospitals in Cameroon, the Truenat MTB Plus assay had a sensitivity of 91% (228/251) to detect TB among those with culture-positive TB; this was similar to the performance of the Xpert MTB/RIF assay to detect TB in this population. Our study provides useful data on the performance of the Truenat MTB Plus assay among people living with HIV, which has not yet been described in other evaluations of this new assay. Among 74 HIV-positive people with culture-positive TB, the sensitivity was 85% (63/74; 95% CI, 75 to 92%) for the Truenat MTB Plus assay and 81% (60/74; 95% CI, 70 to 89%) for the Xpert MTB/RIF assay.

Comparable overall performance of the Truenat MTB Plus with the Xpert MTB/RIF assay was also found in a recent multisite, multicountry evaluation of the Truenat assays (18). In that evaluation, the sensitivity of the Truenat MTB Plus assay was lower than that found here: at reference laboratories, the Truenat MTB Plus had a sensitivity of 85% (95% CI, 81 to 88%). This lower sensitivity may be attributable, at least in part, to the higher proportion of people with smear-negative, culture-positive TB in that population compared to the population studied here (30% versus 19%).

In this evaluation, the overall specificity of the Truenat MTB Plus assay was 96%; 31 of 694 participants with culture-negative results for TB had a result of MTB detected on the Truenat MTB Plus assay. For the purpose of the diagnostic accuracy evaluation, these results are considered as false positives compared to the reference standard of culture. However, following WHO guidance, any result of TB detected by Truenat, Xpert, or culture is considered as bacteriological confirmation of TB, and it is recommended that a person to be evaluated for TB who has bacteriological confirmation of TB should be started on TB treatment (6). While culture is considered the best currently available reference standard, its performance to detect TB depends on a variety of factors, including the method and media used (solid and/or automated liquid culture), the number of specimens cultured per person, the condition of the specimen prior to processing, and the details of the processing method to decontaminate and concentrate the TB bacilli for inoculation. In this study, we cultured specimens on both automated liquid and solid media using standardized protocols, and we cultured two specimens from each participant, for a total of four cultures on two types of media for each participant, which provides a strong reference standard for TB culture. Because the molecular tests detect bacterial DNA, the presence of the DNA detected in specimens from the 31 participants with culture-negative results suggests that these participants produced sputum with TB DNA that was either nonviable or at too low of a concentration to be detected on culture, even when using four cultures per participant. Among these 31 Truenat-positive, culture-negative results, 25 had grades of very low and 6 were graded as low, with DNA detection at a relatively high cycle threshold (*C_T_*) value. TB bacterial DNA may be detected in specimens that are culture-negative for TB and not indicative of current TB disease in people who have a recent history of treatment of TB; in this case, the presence of TB DNA in the sputum is considered to be part of the previous disease process (27). In this study, there were 8 of the 31 people with Truenat-positive, culture-negative results who had a history of TB treatment, and although all but one of these had completed treatment more than 1 year prior to the test (see Table S4 in the supplemental material), it is possible that some of these people had DNA detected in their sputum due to prior rather than current TB disease. The detection of TB DNA in sputum from people with TB culture-negative results has also been described for other molecular assays, including for the Xpert and the Ultra assays (28, 29). The clinical interpretation of these results to determine whether the detection of DNA is linked to current disease would require more intensive clinical follow-up for the participants than we conducted here. Further research is needed to better define preferred patient management for these cases.

Two Truenat assays have been developed for the detection of TB, the Truenat MTB and the Truenat MTB Plus assays. The target sequence of the Truenat MTB assay is the *nrdB* gene (30), and the target sequences of the Truenat MTB Plus assay are the *nrdZ* gene and the multicopy IS*6110* insertion element (21). The inclusion of the insertion element sequences in the MTB Plus assay provides additional sensitivity for TB detection, with the MTB Plus assay having a limit of detection of approximately 30 CFU per mL, while the MTB assay has a limit of detection of approximately 100 CFU/mL as reported by the manufacturer (21, 30). For this analysis, we chose to evaluate the performance of the Truenat MTB Plus assay, due to its higher potential sensitivity and therefore higher perceived value in our setting. The two Truenat assays parallel the two Xpert assays, though with different TB detection mechanisms. The primers in the Xpert MTB/RIF assay amplify portions of the *rpoB* gene, and the primers in the Xpert MTB/RIF Ultra assay amplify portions of the multicopy IS*1081* and IS*6110* insertion elements for TB detection, with reported limits of detection of approximately 113 CFU/mL and 16 CFU/mL, respectively (31). In our study, some specimens that were positive on the Truenat MTB Plus assay and negative on the Xpert MTB/RIF assay were also positive on the Xpert MTB/RIF Ultra assay (Table S4); this is in line with the relatively higher expected sensitivity for the Ultra and Truenat MTB Plus assays compared to the Xpert MTB/RIF assay based on the mechanisms of TB detection. The Truenat MTB Plus and Ultra assays have also demonstrated higher sensitivity compared to the Xpert MTB/RIF assay in a previous study (18).

This study had several strengths. All laboratory testing was performed at an accredited reference laboratory with strong quality control and quality assurance procedures for TB diagnostic testing, as recommended for initial evaluations of new diagnostics (24). For specimen flow, collection of ≥4 mL of sputum and homogenization of the sputum prior to further processing facilitated performance of the index test (Truenat MTB Plus), the comparator test (Xpert), and the reference standard (TB culture) on the same specimen; this is the preferred specimen flow to evaluate assay performance in recent guidance for evaluation of sputum-based tests for TB diagnosis (24). We used a reference standard of four cultures from two specimens collected on 2 days, which provides a relatively robust reference for TB culture, in line with recommendations for culture reference standards from recent systematic reviews (28, 32). We also reported on the identification of culture isolates containing nontuberculous mycobacteria, providing evidence to better evaluate the specificity of this new assay. Truenat MTB Plus testing was performed on each of the two specimens provided by the participants, enabling estimation of the additional sensitivity of the assay when performing a second test for each person to be evaluated for TB. Also, we evaluated the outcomes of all participants for the start of TB treatment and for their vital status at 2 months postenrollment as patient important outcomes.

There were also several limitations to this study. We compared the performance of the Truenat MTB Plus assay with the Xpert MTB/RIF assay but not the Xpert MTB/RIF Ultra assay; because the Ultra is the more sensitive assay of the two, the comparison of the performance Truenat MTB Plus with the Ultra will also be of strong interest for future studies. The study population included only 47 participants with smear-negative, culture-positive TB, so the estimates of assay sensitivity and specificity in this population have wide confidence intervals. In order to perform the index, comparator, and reference standard testing on the same specimen, we only included data from participants who were able to produce two specimens of at least 4 mL in volume; this may have led to the exclusion of people with paucibacillary disease who may be less likely to produce such a relatively large amount of sputum. Also, while participants were enrolled at four sites across Cameroon, most participants (58%) were from one site, and this evaluation includes people from only one country, potentially limiting the generalizability of the results. We did not evaluate the performance of the assay at peripheral laboratories, which are the primary intended setting of use; studies in other settings have evaluated the performance of the assay when performed directly at microscopy centers and hospitals, with good performance reported in these initial studies (14, 18).

In conclusion, the findings reported here provide support for the use of the Truenat MTB Plus assay as a sensitive diagnostic test for TB, including in people living with HIV, as recently recommended by the WHO. Additional information about the interpretation and use of Truenat-positive, culture-negative results are needed to better inform patient management.
