# Supplementary material for: Diagnostic Accuracy of the Truenat MTB Plus Assay and Comparison with the Xpert MTB/RIF Assay to Detect Tuberculosis among Hospital Outpatients in Cameroon
Source: J Clin Microbiol. 2022 Jul 21;60(8):e00155-22. doi: 10.1128/jcm.00155-22 (PMC9383115; doi:10.1128/jcm.00155-22)
Supplement: Supplemental file 1 — Tables S1 to S4. Download jcm.00155-22-s0001.pdf, PDF file, 0.3 MB [file jcm.00155-22-s0001.pdf]

Supplemental material

Table S1. Sensitivity and specificity of the two Truenat MTB Plus tests on two sputum specimens collected on two days, as compared to the reference standard of TB culture. Results for all participants, and by HIV-status, smear status, and history of TB; for a total of 939 participants included in the analysis (248 culture-positive and 691 culture-negative)

**Two Truenat MTB Plus tests on two specimens**	**N**	**TP**	**FP**	**FN**	**TN**	**Sensitivity % (95% CI)**	**Specificity % (95% CI)**
All participants	939	228	45	20	646	92% (88-95%)	93% (91-95%)
HIV status							
HIV+	350	64	16	9	261	88% (77-94%)	94% (91-97%)
HIV-	587	163	29	11	384	94% (89-97%)	93% (90-95%)
Smear status*							
Smear-negative	47	28		19		60% (44-73%)	
Smear-positive	201	200		1		100% (97-100%)	
History of TB							
History of TB	134	22	10	2	100	92% (72-99%)	91% (84-95%)
No history of TB	805	206	35	18	546	92% (87-95%)	94% (92-96%)

[&]There were 6 participants of the total 945 who did not have a 2nd Truenat result recorded, so only 939 participants were included in this analysis; [¶]2 participants did not have HIV status recorded; *Among participants with culture-positive TB

Table S2. Number of participants with one or more culture positive for nontuberculous mycobacteria, by mycobacterial species identified; detailed results by participant are shown in Table S5.

**Species**	**N**
M. abscessus	*6*
M. avium	*5*
M. chelonae	*1*
M. fortuitum	*14*
M. gordonae	*2*
M. interjectum	*1*
M. intracellulare	*12*
M. kansasii	*1*
M. scrofulaceum	*4*
M. simiae	*1*
Other mycobacteria	*12*
**Total**	**59**

Table S3. Result distribution by type of result (valid, invalid or error) obtained for the Truenat MTB Plus assays on the first amplification attempt for each specimen after extraction and the second amplification attempt for those with invalid or error first result; results shown only for Lot B, the second lot received that was used for most of the testing

Result type	***First attempt N, %***		***Second attempt n, %***	
*Error**	*55*	*4.1%*	*13*	*9.3%*
Invalid	*81*	*6.0%*	*17*	*12.1%*
Valid	*1,217*	*89.9%*	*110*	*78.6%*
Total tests	*1,353*		*140*	

**Of the 55 results that were reported as errors, the distribution by type of error as reported by the instrument was: 2 Error-1, 3 Error-1A, 4 Error-2, 45 Error-3, 1 Error-4*

Table S4. Characteristics, laboratory results and clinical outcome for 31 participants with 1st Truenat MTB Plus result positive and TB culture negative results

**Participant characteristics**						**Results- sputum specimen #1**			**Result- sputum specimen #2**	**Outcome**	
SN	Age group (years)	Sex	History of TB	Years since TB treatment	HIV result	Truenat MTB Plus result	Xpert MTB/RIF result	Xpert MTB/RIF Ultra*	Truenat MTB Plus result,	Started TB treatment&	Vital status at 2 months post enrollment
1	35-44	Male	No	N/A	Positive	MTB v. low	NOT	Trace	NOT	No	Alive
2	55-64	Female	No	N/A	Negative	MTB v. low	MTB v. low	--	MTB v. low	Yes	Alive
3	55-64	Female	No	N/A	Positive	MTB v. low	MTB v. low	--	MTB v. low	Yes	Alive
4	45-54	Female	No	N/A	Negative	MTB v. low	NOT	Trace	MTB v. low	No	Alive
5	25-34	Male	Yes	>5 yrs	Positive	MTB v. low	NOT	Trace	--	Yes	Alive
6	55-64	Male	Yes	>2 to <=5 yrs	Negative	MTB low	MTB low	--	NOT	Yes	Alive
7	35-44	Male	Yes	>2 to <=5 yrs	Positive	MTB low	NOT	NOT	NOT	No	Lost to follow-up
8	55-64	Male	Yes	>2 to <=5 yrs	Negative	MTB v. low	NOT	NOT	NOT	Yes	Alive
9	65-74	Female	No	N/A	Negative	MTB low	NOT	NOT	NOT	Yes	Lost to follow-up
10	65-74	Male	No	N/A	Negative	MTB v. low	NOT	NOT	MTB low	No	Dead
11	15-24	Male	Yes	>1 to <=2 yrs	Negative	MTB v. low	NOT	Trace	NOT	No	Lost to follow-up
12	35-44	Female	No	N/A	Negative	MTB v. low	NOT	Trace	NOT	Yes	Alive
13	45-54	Male	No	N/A	Negative	MTB v. low	NOT	Trace	NOT	Yes	Alive
14	25-34	Male	No	N/A	Negative	MTB v. low	NOT	NOT	--	No	Lost to follow-up
15	15-24	Female	No	N/A	Negative	MTB low	NOT	Trace	MTB v. low	Yes	Lost to follow-up
16	75-84	Female	No	N/A	Negative	MTB v. low	NOT	Trace	NOT	No	Alive
17	45-54	Female	No	N/A	Positive	MTB v. low	MTB v. low	--	NOT	Yes	Other
18	55-64	Female	No	N/A	Positive	MTB v. low	NOT	Trace	NOT	Yes	Alive
19	65-74	Male	No	N/A	Negative	MTB v. low	NOT	Trace	NOT	Yes	Dead
20	15-24	Female	No	N/A	Negative	MTB v. low	NOT	NOT	NOT	Yes	Alive
21	55-64	Female	Yes	>1 to <=2 yrs	Positive	MTB v. low	MTB v. low		NOT	Yes	Alive

22	55-64	Female	No	N/A	Negative	MTB low	NOT	NOT	NOT	Yes	Alive
23	35-44	Female	No	N/A	Negative	MTB v. low	NOT	NOT	NOT	No	Alive
24	25-34	Male	No	N/A	Positive	MTB low	NOT	Trace	MTB v. low	No	Lost to follow-up
25	55-64	Female	No	N/A	Positive	MTB v. low	NOT	Trace	MTB v. low	Yes	Alive
26	35-44	Female	No	N/A	Negative	MTB v. low	MTB v. low	--	NOT	Yes	Alive
27	55-64	Female	No	N/A	Negative	MTB v. low	NOT	NOT	MTB v. low	No	Dead
28	35-44	Male	Yes	>5 yrs	Positive	MTB v. low	NOT	Trace	NOT	Yes	Alive
29	45-54	Male	No	N/A	Positive	MTB v. low	NOT	NOT	NOT	Yes	Alive
30	65-74	Male	No	N/A	Negative	MTB v. low	MTB v. low	--	NOT	Yes	Alive
31	45-54	Male	Yes	<=1 yr	Negative	MTB v. low	MTB v. low	--	MTB v. low	Yes	Alive

All participants provided two sputum specimens that were smear-negative and culture-negative for TB.
MTB- Mycobacterium tuberculosis complex; NOT- MTB not detected
*The Xpert MTB/RIF Ultra assay produces one of 5 results when MTB is detected; these are high, medium, low, very low or trace. The trace result indicates that *rpoB* gene amplification was not detected and only the multi-copy insertion elements were detected on amplification.
-- No testing done. Ultra testing was only performed for specimens with Xpert MTB/RIF results that were negative..; 2 people did not have a second Truenat test result recorded.
[&]Reasons for not starting TB treatment varied by participant; in some cases, the clinician recommended not starting or not continuing TB treatment and in other cases the participant declined TB treatment or was lost to follow-up before being offered treatment.
